# Signs of Cardiac Autonomic Imbalance and Proarrhythmic Remodeling in FTO Deficient Mice

**DOI:** 10.1371/journal.pone.0095499

**Published:** 2014-04-17

**Authors:** Luca Carnevali, Gallia Graiani, Stefano Rossi, Mumna Al Banchaabouchi, Emilio Macchi, Federico Quaini, Nadia Rosenthal, Andrea Sgoifo

**Affiliations:** 1 Department of Neuroscience, University of Parma, Parma, Italy; 2 Department of Clinical and Experimental Medicine, University of Parma, Parma, Italy; 3 Department of Life Sciences, University of Parma, Parma, Italy; 4 Preclinical Phenotyping Facility, CSF-Campus Science Support Facilities GmbH, Vienna, Austria; 5 Australian Regenerative Medicine Institute/EMBL Australia, Monash University, Melbourne, Victoria, Australia; 6 European Molecular Biology Laboratory (EMBL) Mouse Biology Unit, Monterotondo, Italy; University of Adelaide, Australia

## Abstract

In humans, variants of the fat mass and obesity associated (FTO) gene have recently been associated with obesity. However, the physiological function of FTO is not well defined. Previous investigations in mice have linked FTO deficiency to growth retardation, loss of white adipose tissue, increased energy metabolism and enhanced systemic sympathetic activation. In this study we investigated for the first time the effects of global knockout of the mouse FTO gene on cardiac function and its autonomic neural regulation. ECG recordings were acquired via radiotelemetry in homozygous knockout (n = 12) and wild-type (n = 8) mice during resting and stress conditions, and analyzed by means of time- and frequency-domain indexes of heart rate variability. In the same animals, cardiac electrophysiological properties (assessed by epicardial mapping) and structural characteristics were investigated. Our data indicate that FTO knockout mice were characterized by (i) higher heart rate values during resting and stress conditions, (ii) heart rate variability changes (increased LF to HF ratio), (iii) larger vulnerability to stress-induced tachyarrhythmias, (iv) altered ventricular repolarization, and (v) cardiac hypertrophy compared to wild-type counterparts. We conclude that FTO deficiency in mice leads to an imbalance of the autonomic neural modulation of cardiac function in the sympathetic direction and to a potentially proarrhythmic remodeling of electrical and structural properties of the heart.

## Introduction

The last few years have witnessed a surge of research on the study of the physiological function and *in vivo* substrates of the fat mass and obesity associated (FTO) gene (for a review see [1]). Recent interest in the FTO gene stems from studies demonstrating an association between a single nucleotide polymorphism in the first intron of the gene with obesity-related traits and higher obesity risk in different human populations [2]–[5].

From a molecular point of view, FTO has been characterized as a 2-oxogluterate dependent dioxygenase that is involved in nucleic acid modification [6]. In mice, global deletion of FTO has been linked to postnatal growth retardation [7], reduction in adipose tissue [8], reduction in lean mass [8], [9] and increased energy expenditure [8], [9], thus supporting the involvement of FTO in energy metabolism and body weight regulation.

FTO is ubiquitously expressed. In the brain, strong expression is seen in the hippocampus, cerebellum and hypothalamus [6], [7], [10]. The hypothalamic expression of FTO suggests a potential role of this gene in the regulation of autonomic function [11], [12]. The paraventricular and dorsomedial nuclei of the hypothalamus, which show particularly high expression of FTO, are key modulators of sympathetic outflow [11], [12]. Interestingly, preliminary evidence seems to connect FTO deficiency in mice with increased sympathetic nervous system activity [13]. FTO is also expressed in many other tissues including the heart, albeit at substantially lower levels [6], [7].

Given these considerations, the principal objective of the present study was to investigate the potential role of the FTO gene in the autonomic neural regulation of cardiac function, by means of a mouse model of FTO deficiency. Based on the above observations, we hypothesized that global knockout of FTO would lead to an increased sympathetic excitation of the heart. To test this hypothesis, sympathetic and parasympathetic (vagal) influences on the heart were assessed during resting and stress conditions via time- and frequency-domain analysis of heart rate variability (HRV). We also evaluated whether supposed cardiac sympathetic hyperactivity in FTO knockout mice was associated with increased arrhythmia vulnerability, and investigated potential mediating mechanisms at the electrical and structural level of the heart.

## Methods

### Ethics Statement and Animals

All experimental procedures and protocols were approved by the Veterinarian Animal Care and Use Committee of Parma University and conducted in accordance with the European Community Council Directives of 22 September 2010 (2010/63/UE). Experiments were performed on 4-month-old male homozygous knockout (Fto^−/−^, n = 12) and wild-type (Fto^+/+^, n = 8) mice obtained from the Mouse Biology Unit of the European Molecular Laboratory of Monterotondo, where they had been generated from crosses between heterozygous animals. Fto^−/−^ mice were created using homologous recombination as described previously in detail [8] and maintained on original C57BL/6N background. At their arrival in our laboratory, mice were housed in groups of 3–4 per cage and kept at ambient temperature of 22–24°C and on a reversed 12∶12 light-dark cycle (light on at 19∶00 h), with food and water *ad libitum*.

### Radiotelemetry System

A radiotelemetry system (Data Sciences International, St. Paul, MN, USA) was used for recording ECG (sampling rate 2 kHz), temperature and activity (sampling rate 256 Hz) signals. It consisted of wireless transmitters (TA10ETA-F20) and platform receivers (RPC-1), which were controlled by ART-Gold 1.10 data acquisition system. The transmitters were implanted in experimental mice according to a procedure described by Sgoifo and colleagues [14]. The surgery was performed under isoflurane (2% in 100% oxygen) anesthesia. The transmitter body was placed in the abdominal cavity; one electrode was fixed to the dorsal surface of the xyphoid process and the other electrode was placed in the anterior mediastinum close to the right atrium. Such electrode location guarantees high-quality ECG recordings, even during vigorous physical activity. Immediately after surgery, mice were individually housed, injected for 2 days with Gentamicin sulphate (Aagent, Fatro, 0.2 mg/kg, s.c.) and allowed 10 days of recovery before the start of experimental recordings.

### General Experimental Outline

Following recovery from surgery, mice were left undisturbed in their home cages for 5 days for collection of baseline daily rhythms of heart rate (HR, expressed as beats per minute (bpm)), temperature (T, °C) and locomotor activity (LOC, expressed as counts per minute (cpm)). Subsequently, rats were submitted on different days to: i) two acute stress challenges, namely injection of saline (day 1) and restraint test (day 4); ii) epicardial mapping (day 7). These tests were carried out between 10∶00 and 14∶00 (i.e., the dark phase of the light/dark cycle). At sacrifice, the hearts were excised for structural and morphological analyses. Specific experimental procedures and data analysis are described in the following sections.

### Baseline Daily Rhythms

ECG, T and LOC were sampled around-the-clock for 2 minutes every hour over a period of 5 days for collection of baseline daily rhythms. Data analysis was performed as follows. Separate estimates of HR, T and LOC were initially generated for each 2-min recording period and subsequently averaged as mean values of 12 h-light and 12 h-dark daily phases. These parameters were then further averaged as means of the 5 days of the light and dark phases.

### Acute Stress Challenges

On day 1, Fto^+/+^ and Fto^−/−^ mice received a subcutaneous injection of saline (0.9% NaCl, vol: 1 ml/kg). Continuous ECG recordings were performed prior to (30 min, baseline conditions) and following (60 min) the injection, with the mice in their home cages. On day 4, Fto^+/+^ and Fto^−/−^ mice were placed for 15 min in a cylindrical plastic restrainer fitted closely to the body size (inner diameter 4 cm; length 12 cm) and closed at both ends by removable partitions with holes for air circulation. Continuous ECG recordings were performed prior to the test (30 min, with the mice in their home cages (baseline conditions)), during the restraint test (15 min) and throughout the recovery period (45 min, with the mice in their home cages).

Data analysis was conducted as follows. Initially, we split each recording period in 3-min epochs (0–3 min, 3–6, etc.). For each epoch, separate estimates of HR, HRV indexes, T and LOC were generated. Time- and frequency-domain parameters of HRV were quantified using ChartPro 5.0 software (ADInstruments, Sydney, Australia), following the guidelines suggested by Thireau and colleagues for the assessment of HRV parameters in mice [15]. In the time-domain, we obtained the root mean square of successive R-R interval differences (RMSSD, ms), which estimates the activity of the parasympathetic nervous system [16]. For spectral (frequency-domain) analysis of HRV, a power spectrum was obtained with a fast Fourier transform-based method (Welch’s periodogram: 256 points, 50% overlap, and Hamming window). We considered: i) the total power of the spectrum (ms^2^), which reflects all the cyclic components responsible for variability, ii) the power (ms^2^) of the low frequency band (LF, 0.15–1.5 Hz), which is a non-specific index as it contains contributions of both the sympathetic and parasympathetic influences [17], iii) the power (ms^2^) of the high frequency band (HF; 1.5–5.0 Hz), which is due to the activity of the parasympathetic nervous system and includes respiration-linked oscillations of HR [18], and iv) the low frequency/high frequency ratio (LF/HF), which estimates the fractional distribution of power and is taken as a synthetic measure of sympathovagal balance [19].

In addition, ECG signals obtained during baseline, pre-saline-injection recordings were further analyzed as follows. Three 2s-segments of high ECG quality [20] were randomly selected for each 3-min epochs in order to quantify the duration of: i) P wave; ii) PQ segment; iii) QRS complex; iv) QTc, which is the QT interval normalized to cycle length.

Lastly, the occurrence of arrhythmic events was determined and quantified off-line based on the Lambeth Conventions for the study of experimental arrhythmias [21]. We determined and quantified the separate occurrence of supraventricular (SV) and ventricular (V) ectopic beats and the total number of tachyarrhythmic events in baseline and challenge conditions.

### Epicardial Mapping

On day 7, mice were anesthesized with Xylazine (10 mg/kg, i.p.) and Ketamine (50 mg/kg, i.p.). Subsequently, the heart was exposed through a longitudinal sternotomy. An epicardial electrode array (5×5 row and column with a 0.6 mm resolution square mesh) was used to record unipolar epicardial electrograms during sinus rhythm and ventricular pacing in order to determine cardiac excitability, conduction velocity of the electrical impulse, and refractoriness [22]. The epicardial mapping protocol was prematurely interrupted in two Fto^+/+^ and three Fto^−/−^ mice because of technical difficulties that precluded accurate recording. Therefore, data analysis was conducted in 6 Fto^+/+^ and 9 Fto^−/−^ mice as follows.

#### i) Excitability

The strength-duration curve was obtained as a measure of cardiac excitability [23] at 5 selected electrodes of the array, as described previously in detail [22]. The strength duration curve is represented by the equation I = Rh(1+Chr/T), where I is the threshold current strength, T is the pulse duration, Rh is the rheobase (i.e., the lowest intensity with infinite pulse duration which succeeds in eliciting a propagated response in excitable tissues), and Chr is the chronaxie (i.e., the pulse duration having a threshold twice that of Rh).

#### ii) Conduction velocity

Activation sequences (isochrone maps) were computed from the activation times of paced beats using custom written software, and conduction velocity longitudinally and conduction velocity transversally to fiber orientation were calculated from them, as previously described [22].

#### iii) Refractoriness

Ten baseline stimuli (S1), 1 ms width and twice diastolic threshold intensity, were delivered at each of the 5 selected electrodes of the array at a frequency slightly higher than basal cycle length, as in [22]. The S1 pacing sequence was followed by an extra-stimulus (S2, four-fold S1 intensity) whose delay from previous S1 was first progressively decremented by 10 ms steps until capture was lost and then progressively incremented by 2 ms steps till capture was resumed. We considered: i) the effective refractory period (ERP), which was defined as the shortest S1–S2 time interval at which excitation from S2 failed, and ii) the spatial dispersion of the ERP, measured as the maximum difference (range) and the standard deviation (SD) of the mean [24].

### Post Mortem Measurements

Upon completion of the epicardial mapping, the heart was arrested in diastole by cadmium chloride solution injection (100 mM, i.v.). The heart of the 6 Fto^+/+^ and 9 Fto^−/−^ mice that concluded the mapping protocol was removed from the chest and fixed in 10% buffered formalin solution.

#### i) Cardiac anatomy

After 24 h, the free walls of the right ventricle (RV) and the left ventricle (LV) inclusive of interventricular septum were separated and their weights recorded. These data and heart weight (HW) were normalized to body weight (BW) value.

*ii)* Transverse sections of the LV were paraffin embedded, 5-µm thick sections were then cut and stained with Haemtoxylin & Eosin or Masson’s Trichrome following procedures that have been described previously in detail [25], [26] in order to evaluate: i) the volume fraction of myocytes, ii) the total amount of fibrosis, and iii) interstitial extension. Specifically, the number of points overlying each tissue components was counted and expressed as percentage of the total number of points explored. All these morphometric measurements were obtained with the aid of a grid defining a tissue area of 0.23 mm^2^ and containing 42 sampling points each covering an area of 0.0052 mm^2^.

### Statistics

All statistical analyses were performed using the software package SPSS (version 20). Two-way ANOVA for repeated measures with group as between-subject factor (2 levels: Fto^+/+^ and Fto^−/−^ ) was applied for data obtained from: i) baseline daily rhythms, with time as within-subject factor (2 levels: light and dark phases); ii) injection of saline, with time as within-subject factor (4 levels: baseline; post-injection 1, 2, and 3); iii) restraint test, with time as within-subject factor (5 levels: baseline; test; recovery 1, 2, and 3). Follow-up analyses were conducted using Student’s “t” tests, with a Bonferroni correction for multiple comparisons for each outcome variable separately. A priori Student’s “t”-tests, after controlling for homogeneity of variance via Levene test, were applied for comparisons between Fto^+/+^ and Fto^−/−^ mice on: i) the occurrence of arrhythmic events; ii) data obtained from epicardial mapping; iii) measurements at sacrifice. Data are presented as means ± standard error of the mean (SEM). Statistical significance was set at p<0.05.

## Results

### Baseline Daily Rhythms

**Figure 1:**
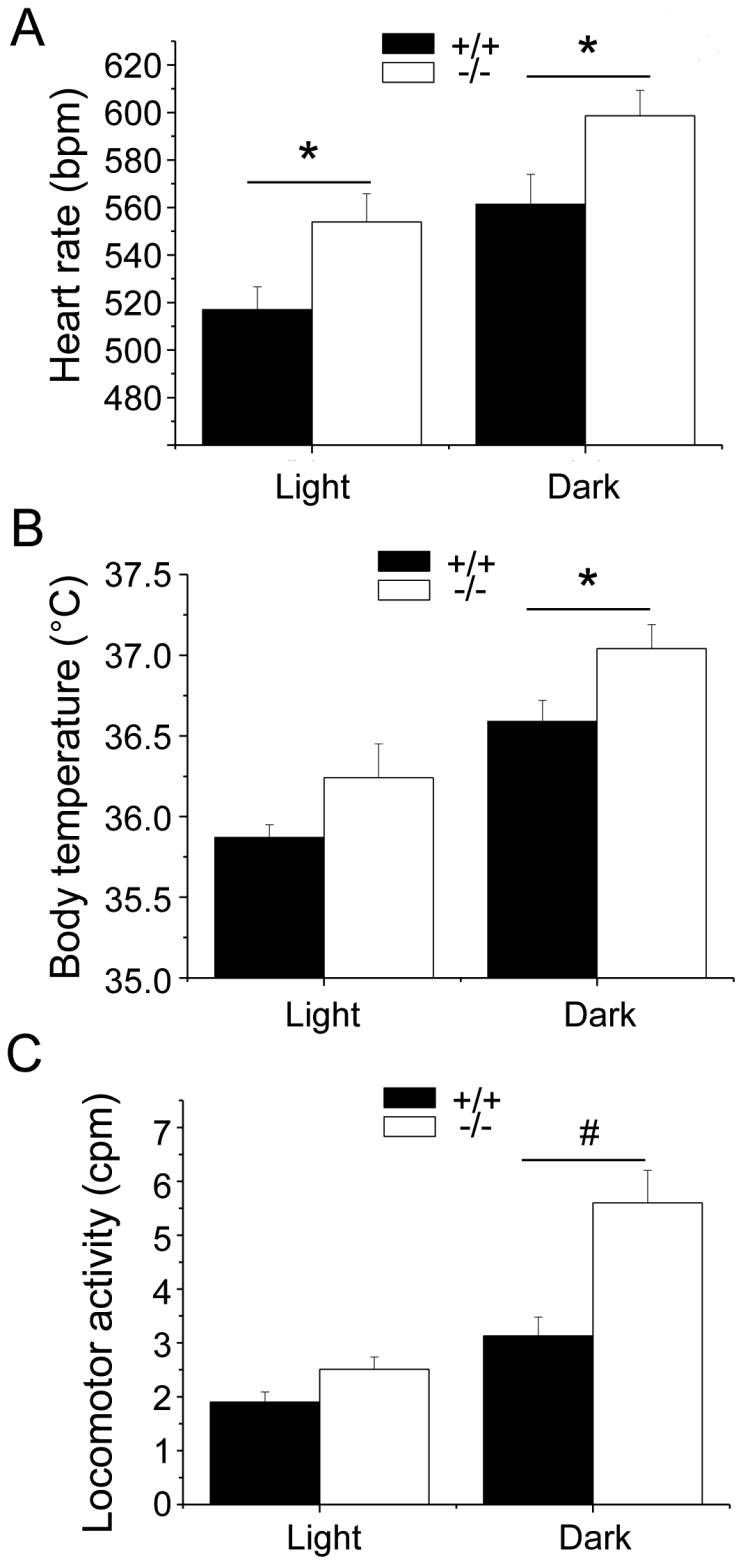
Daily rhythms of heart rate, body temperature and locomotor activity. For the 12-light and 12 h-dark phases, values are reported as means ± SEM of data obtained by averaging multiple 2-min segments acquired every hour over a period of 5 days in Fto^+/+^ (n = 8) and Fto^−/−^ (n = 12) mice. * and ^#^ indicate a significant difference between Fto^+/+^ and Fto^−/−^ mice (p<0.05 and p<0.01, respectively).

The daily rhythms of HR, T and LOC in wild-type and Fto^−/−^ mice under during resting conditions are depicted in Figure 1. Two-way ANOVA yielded a significant effect of i) group on HR (F = 5.2, p<0.05) and LOC (F = 11.3, p<0.01) values and ii) time on HR (F = 150.3, p<0.01), T (F = 96.7, p<0.01) and LOC (F = 49.4, p<0.01) values.

Fto^−/−^ mice had significantly higher values of HR than Fto^+/+^ counterparts during both the light (t = 2.2, p<0.05) and the dark (t = 2.2, p<0.05) phases of the circadian cycle (Figure 1A). In addition, Fto^−/−^ mice had higher values of T than Fto^+/+^ counterparts, although statistical significance was reached only during the dark phase (t = 2.1, p<0.05) (Figure 1B). Likewise, Fto^−/−^ mice exhibited higher values of LOC than Fto^+/+^ counterparts during the dark phase (t = 3.5, p<0.01), whereas during the light phase the two groups showed similar LOC values (Figure 1C).

### Injection of Saline

**Figure 2:**
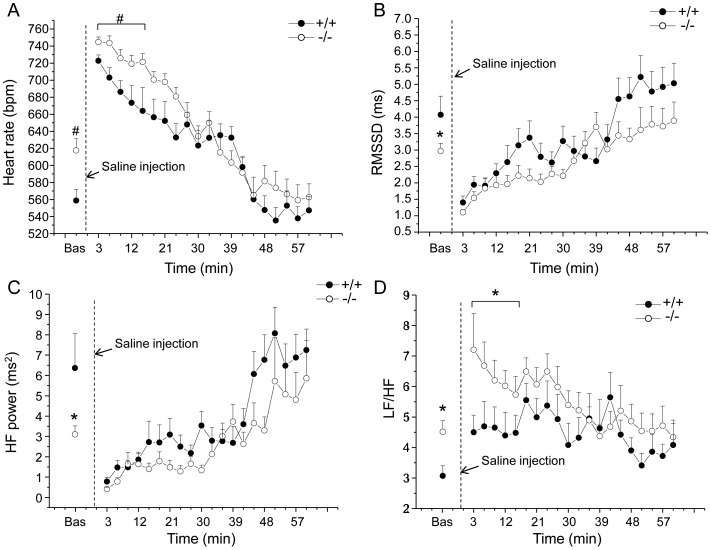
Cardiac autonomic response to the injection of saline. Time course of changes in heart rate (panel A), RMSSD values (panel B), high frequency (HF) spectral power (panel C) and LF to HF ratio (panel D) following the injection of saline, in Fto^+/+^ (n = 8) and Fto^−/−^ (n = 12) mice. Baseline reference value (bas) is the mean value of the ten 3-min time points in resting conditions. Values are expressed means ± SEM. * and ^#^ indicate a significant difference between Fto^+/+^ and Fto^−/−^ mice (p<0.05 and p<0.01, respectively).

Cardiac autonomic responses to the injection of saline are depicted in Figure 2 and detailed in Table 1. Two-way ANOVA yielded: i) significant effect of group on HR (F = 5.0, p<0.05) and LF/HF (F = 4.3, p = 0.05) values, ii) significant effect of time on HR (F = 27.8, p<0.01), RMSSD (F = 15.8, p<0.01), LF (F = 13.4, p<0.01), HF (F = 5.3, p<0.05) and LOC (F = 10.6, p<0.01) values and iii) a time × group interaction on T values (F = 4.9, p<0.05).

**Table 1:** Radiotelemetric and HRV parameters in response to the saline injection test.

		Basal	Post-Injection(min 0–15)	Post-Injection(min 15–30)	Post-Injection(min 30–45)	Post-Injection(min 45–60)
HR (bpm)	+/+	559±13	690±13	642±16	612±8	544±15
	−/−	618±14^#^	731±7^#^	675±9	605±14	568±17
Total power (ms^2^)	+/+	55.8±11.5	21.1±5.6	32.8±6.1	40.3±6.1	55.6±9.2
	−/−	39.2±5.1	16.6±3.8	19.4±3.6	38.4±8.2	47.7±9.0
RMSSD (ms)	+/+	4.1±0.6	2.0±0.2	3.0±0.4	3.3±0.4	4.9±0.6
	−/−	3.0±0.2*	1.7±0.2	2.2±0.2	3.2±0.3	3.7±0.5
HF power (ms^2^)	+/+	6.4±1.7	1.7±0.3	2.8±0.5	3.6±0.6	7.1±1.1
	−/−	3.1±0.4*	1.2±0.3	1.5±0.3	3.0±0.6	5.0±1.4
LF power (ms^2^)	+/+	19.3±4.6	7.2±1.5	14.1±3.1	17.7±3.6	28.2±5.9
	−/−	13.1±1.7	7.2±1.7	9.2±1.4	13.7±2.4	16.6±3.6
LF/HF	+/+	3.1±0.3	4.4±0.6	4.9±0.7	4.6±0.6	3.7±0.4
	−/−	4.5±0.4*	6.2±0.6*	6.1±0.4	4.5±0.4	4.4±0.6
T (°C)	+/+	36.4±0.1	36.8±0.2	37.1±0.1	37.0±0.2	36.8±0.1
	−/−	36.8±0.1*	37.2±0.1	37.4±0.1	37.0±0.1	36.7±0.1
LOC (cpm)	+/+	2.3±0.5	11.5±2.7	2.3±0.9	4.6±2.2	3.7±1.2
	−/−	4.2±1.3	12.4±2.1	5.5±1.8	3.2±1.3	2.8±1.2

Values are reported as means ± SEM of data obtained by averaging multiple 3-min segments acquired in baseline conditions (30 min) and following the injection of saline (60 min) in Fto^+/+^ (n = 8) and Fto^−/−^ mice (n = 12). Abbreviations: HRV = heart rate variability; HR = heart rate; RMSSD = square root of the mean squared differences of successive RR intervals; HF = high-frequency; LF = low-frequency; T = body temperature; LOC = locomotor activity. * and ^#^ indicate a significant difference between Fto^+/+^ and Fto^−/−^ mice (p<0.05 and p<0.01, respectively).

Before the test, baseline HR was significantly higher in Fto^−/−^ than in Fto^+/+^ mice (t = 2.9, p<0.01) (Figure 2A and Table 1). In the same period, HRV analysis revealed i) significantly lower values of RMSSD (t = −2.2, p<0.05) and HF spectral power (t = −2.2, p<0.05) in Fto^−/−^ mice compared to Fto^+/+^ counterparts (Figure 2B, C and Table 1) and ii) significantly higher LF to HF ratio in Fto^−/−^ than in Fto^+/+^ mice (t = 2.7, p<0.05) (Figure 2D and Table 1).

During the first 15 min that followed the injection of saline, mean HR was significantly higher in Fto^−/−^ than in Fto^+/+^ mice (t = 2.9, p<0.01) (Figure 2A and Table 1). In the same period, no differences were found in RMSSD and HF spectral power values between the two groups (Figure 2B, C and Table 1). However, LF to HF ratio resulted significantly higher in Fto^−/−^ than in Fto^+/+^ mice (t = 2.1, p<0.05) (Figure 2D and Table 1).

**Figure 3:**
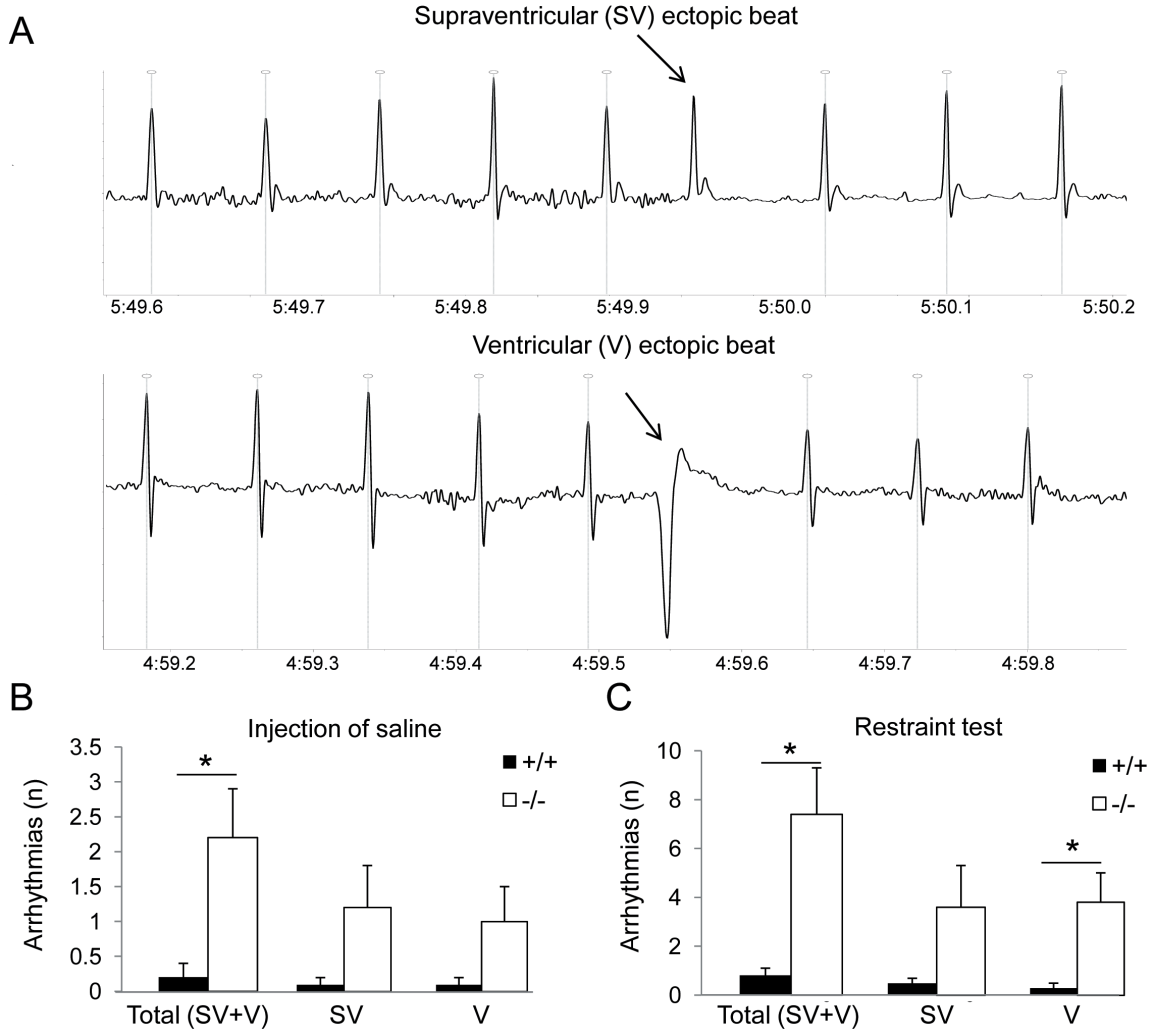
Susceptibility to cardiac tachyarrhythmias. Panel A shows an example of ECG traces belonging to a representative Fto^−/−^ mouse with isolated supraventricular (SV) and ventricular (V) ectopic beats. Panels B and C report the incidence of tachyarrhythmias following the injection of saline and during the restraint test, respectively, in Fto^+/+^ (n = 8) and Fto^−/−^ (n = 12) mice. Values are reported as means ± SEM. * indicates a significant difference between Fto^+/+^ and Fto^−/−^ mice (p<0.05).

In both groups, the total incidence of tachyarrhytmic events during baseline recordings was almost null (Fto^−/−^ = 0.5±0.3 events vs. Fto^+/+^ = 0.9±0.0 events). Following the injection of saline, the total incidence of tachyarrhthmic events was significantly larger in Fto^−/−^ mice compared to Fto^+/+^ counterparts (t = 2.1, p<0.05) (Figure 3B).

### Restraint Test

**Figure 4:**
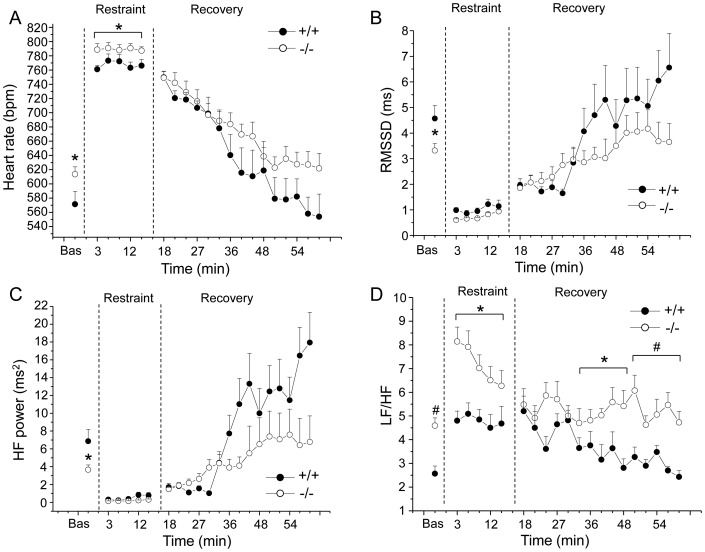
Cardiac autonomic response to restraint test. Time course of changes in heart rate (panel A), RMSSD values (panel B), high frequency (HF) spectral power (panel C), and LF to HF ratio (panel D) during the restraint test and the recovery phase, in Fto^+/+^ (n = 8) and Fto^−/−^ (n = 12) mice. Baseline reference value (bas) is the mean value of the ten 3-min time points in resting conditions. Values are expressed as means ± SEM. * and ^#^ indicate a significant difference between Fto^+/+^ and Fto^−/−^ mice (p<0.05 and p<0.01, respectively).

Cardiac autonomic responses to the restraint test are depicted in Figure 4 and detailed in Table 2. Two-way ANOVA yielded significant effects of i) group on HR (F = 4.4, p = 0.05) and LF/HF (F = 17.6, p<0.01) values and ii) time on HR (F = 13.9, p<0.01), RMSSD (F = 12.7, p<0.01), total power (F = 18.3, p<0.01), LF (F = 28.2, p<0.01) and HF (F = 16.8, p<0.01) values.

**Table 2:** Radiotelemetric and HRV parameters in response to the restraint test.

		Basal	Restraint	Recovery (min 0–15)	Recovery (min 15–30)	Recovery (min 30–45)
HR (bpm)	+/+	571±18	767±8	719±10	633±29	570±27
	−/−	614±11*	789±6*	727±14	669±15	627±16
Total power (ms^2^)	+/+	59.8±13.4	5.7±1.9	23.8±4.3	72.6±16.1	90.6±18.5
	−/−	47.6±7.9	3.4±0.4	28.9±5.2	46.3±9.9	52.3±17.3
RMSSD (ms)	+/+	4.6±0.5	1.0±0.1	1.9±0.2	4.2±1.0	5.7±1.2
	−/−	3.3±0.3*	0.7±0.1	2.2±0.3	3.1±0.5	3.9±0.7
HF power (ms^2^)	+/+	6.9±1.3	0.5±0.2	1.5±0.2	9.3±2.2	14.2±2.9
	−/−	3.7±0.6*	0.2±0.1	2.4±0.5	4.7±1.9	7.1±3.6
LF power (ms^2^)	+/+	17.4±3.0	2.4±0.8	7.2±1.3	26.6±5.0	36.2±6.8
	−/−	15.9±2.2	1.3±0.2	12.4±2.5	18.6±5.0	26.5±8.7
LF/HF	+/+	2.6±0.3	4.7±0.5	4.6±0.5	3.3±0.6	2.8±0.2
	−/−	4.6±0.4^#^	6.7±0.7*	5.5±0.6	5.0±0.5*	5.1±0.6^#^
T (°C)	+/+	36.7±0.1	37.1±0.3	37.5±0.2	37.1±0.1	36.7±0.2
	−/−	36.7±0.1	37.5±0.2	37.9±0.2	37.5±0.2	37.1±0.3
LOC (cpm)	+/+	1.6±0.5	5.7±1.3	13.2±2.9	3.9±1.8	3.3±1.8
	−/−	3.4±0.7	4.6±0.7	16.5±2.5	6.4±2.0	5.1±2.0

Values are reported as means ± SEM of data obtained by averaging multiple 3-min segments acquired in baseline conditions (30 min), during the restraint (15 min) and the recovery phase (45 min) in Fto^+/+^ (n = 8) and Fto^−/−^ mice (n = 12). Abbreviations: HRV = heart rate variability; HR = heart rate; RMSSD = square root of the mean squared differences of successive RR intervals; HF = high-frequency; LF = low-frequency; T = body temperature; LOC = locomotor activity. * and ^#^ indicate a significant difference between Fto^+/+^ and Fto^−/−^ mice (p<0.05 and p<0.01, respectively).

Before the test, baseline HR was significantly higher in Fto^−/−^ than in Fto^+/+^ mice (t = 2.1, p<0.05) (Figure 4A and Table 2). In the same period, HRV analysis revealed i) significantly lower values of RMSSD (t = −2.3, p<0.05) and HF spectral power (t = −2.4, p<0.05) in Fto^−/−^ mice compared to Fto^+/+^ counterparts (Figure 4B, C and Table 2) and ii) significantly higher LF to HF ratio in Fto^−/−^ than in Fto^+/+^ mice (t = 4.2, p<0.01) (Figure 4D and Table 2).

During the test, mean HR was significantly higher in Fto^−/−^ than in Fto^+/+^ mice (t = 2.2, p<0.05) (Figure 4A and Table 2). In the same period, no differences were found in RMSSD and HF spectral power values between the two groups (Figure 4B, C and Table 2). However, LF to HF ratio resulted significantly higher in Fto^−/−^ than in Fto^+/+^ mice (t = 2.3, p<0.05) (Figure 4D and Table 2). During the recovery phase, no differences between Fto^−/−^ and Fto^+/+^ mice were found in mean HR (Figure 4A and Table 2), as well as in RMSSD and HF spectral power values (Figure 4B, C and Table 2). However, LF to HF ratio resulted significantly higher in Fto^−/−^ than in Fto^+/+^ mice during the second (t = 2.3, p<0.05) and third (t = 3.8, p<0.01) 15-min recording period (Figure 4D and Table 2).

In both groups, the total incidence of tachyarrhytmic events during baseline recordings was almost null (Fto^−/−^ = 0.5±0.3 events vs. Fto^+/+^ = 0.6±0.4 events). During the restraint test, the total incidence of tachyarrhytmic events was significantly larger in Fto^−/−^ mice compared to Fto^+/+^ counterparts (t = 2.9, p<0.05) (Figure 3C).

### Cardiac Intervals

**Figure 5:**
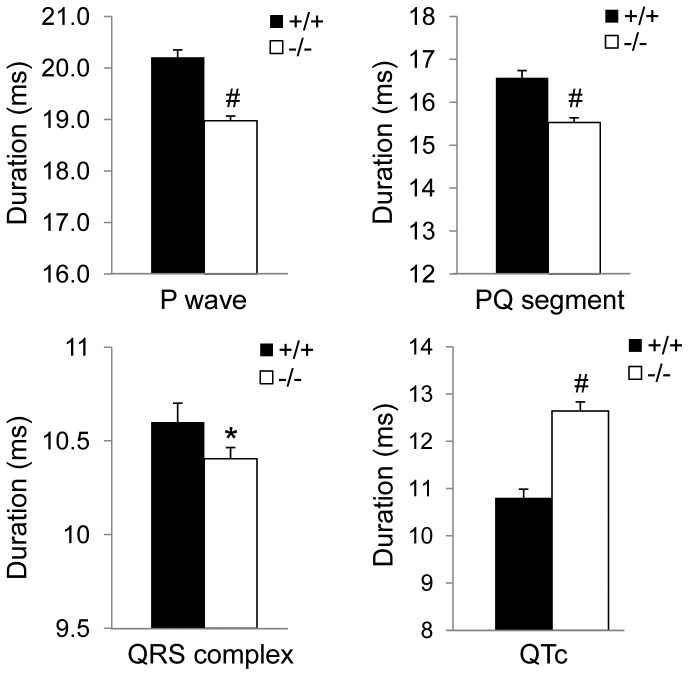
Cardiac interval duration. Values are expressed as means ± SEM. * and ^#^ indicate a significant difference between Fto^+/+^ (n = 8) and Fto^−/−^ (n = 12) mice (p<0.05 and p<0.01, respectively).

The duration of P wave and PQ segment was significantly shorter in Fto^−/−^ mice compared to Fto^+/+^ counterparts (P wave: t = −7.8, p<0.01; PQ segment: t = −5.4, p<0.01) (Figure 5). Likewise, QRS complex duration was significantly shorter in Fto^−/−^ than in Fto^+/+^ mice (t = −2.1, p<0.05) (Figure 5). On the other hand, the duration of QTc was significantly longer in Fto^−/−^ mice compared to Fto^+/+^ counterparts (t = 6.5, p<0.01) (Figure 5).

### Epicardial Mapping

#### Excitability

Rheobase and chronaxie values, which were determined from the strength-duration curve, were similar between Fto^−/−^ and Fto^+/+^ mice (rheobase: Fto^−/−^ = 31.4±5.0 µA vs. Fto^+/+^ = 31.4±2.7 µA; chronaxie: Fto^−/− = ^1.6±0.2 ms vs. Fto^+/+^ = 1.6±0.2 ms).

#### Conduction velocity

Longitudinal ventricular conduction velocity was significantly faster in the heart of Fto^−/−^ mice compared with the heart of Fto^+/+^ mice (Fto^−/−^ = 0.511±0.003 m/s vs. Fto^+/+^ = 0.486±0.006 m/s, t = 3.92, p<0.01), whereas no differences in transversal ventricular conduction velocity were observed between the two groups (Fto^−/−^ = 0.273±0.002 m/s vs. Fto^+/+^ = 0.268±0.004 m/s).

#### Refractoriness

The duration of the ERP was similar between Fto^−/−^ and Fto^+/+^ mice (Fto^−/−^ = 82.1±2.3 ms vs. Fto^+/+^ = 79.5±3.1 ms). Likewise, the spatial dispersion of the ERP was similar between Fto^−/−^ and Fto^+/+^ mice (range: Fto^−/−^ = 26.0±4.0 ms vs. Fto^+/+^ = 28.0±4.5 ms; SD: Fto^−/−^ = 11.3±1.9 vs. Fto^+/+^ = 11.7±1.7).

### Measurements at Sacrifice

Before euthanasia, Fto^−/−^ and Fto^+/+^ mice had similar BW (Fto^−/−^ = 29.6±0.7 g vs. Fto^+/+^ = 31.0±1.0 g).

#### Cardiac anatomy

HW and HW corrected for BW (HW/BW ratio) were significantly higher in Fto^−/−^ than in Fto^+/+^ mice (HW: t = −2.7, p<0.05; HW/BW: t = −3.3, p<0.01) (Table 3). Likewise, LVW, RVW, and their values corrected for BW were significantly augmented in Fto^−/−^ compared to Fto^+/+^ mice (LVW: t = −2.2, p<0.05; RVW: t = −2.2, p<0.05; LVW/BW: t = −3.1, p<0.01; RVW/BW: t = −2.4, p<0.05) (Table 3).

**Table 3:** Gross cardiac characteristics.

	Fto^+/+^ (n = 6)	Fto^−/−^ (n = 9)
HW (mg)	121±6	160±11*
HW/BW (mg/g)	3.91±0.17	5.40±0.34^#^
LVW (mg)	85±5	104±6*
LVW/BW (mg/g)	2.73±0.15	3.50±0.18^#^
RVW (mg)	25±1	35±4*
RVW/BW (mg/g)	0.80±0.05	1.18±0.13*

Values are reported as means ± SEM. Abbreviations: HW = heart weight; BW = body weight; LVW = left ventricular weight; RVW = right ventricular weight. * and ^#^ indicate a significant difference between Fto^+/+^ and Fto^−/−^ mice (p<0.05 and p<0.01, respectively).

#### Tissue morphometry

Morphometric analysis did not show significant changes in the volume fraction of myocytes (Fto^−/−^ = 90.8±1.4% vs. Fto^+/+^ = 89.9±1.5%) and interstitial compartments (Fto^−/−^ = 9.0±1.5% vs. Fto^+/+^ = 9.8±1.5%). Myocardial fibrosis was negligible in the LV myocardium of Fto^−/−^ and Fto^+/+^ mice (Fto^−/−^ = 0.13±0.08% vs. Fto^+/+^ = 0.26±0.10%).

## Discussion

The major and novel finding of this study is that FTO deficiency in mice leads to increased heart rate in resting and stress conditions. Such positive chronotropic effect appeared to be linked to a shift of the autonomic balance towards a sympathetic prevalence and was associated with: (i) potentially proarrhythmic remodeling at the electrical (altered ventricular repolarization) and structural (hyperthrophy) level of the heart and (ii) increased vulnerability to stress-induced arrhythmias.

A previous study in a mouse model bearing a missense mutation in the FTO gene provided preliminary evidence linking FTO deficiency to increased sympathetic nervous system activity (measured by urinary noradrenaline levels) [13]. However, to the best of our knowledge, this study is the first description of the effects of global knockout of FTO on cardiac function and its autonomic neural regulation.

In resting conditions, FTO deficient mice were characterized by higher heart rate values than wild-type mice, both during the active (dark) and inactive (light) phase of the daily cycle. Likewise, we found signs of elevated body temperature in mice lacking the FTO gene. Clearly, differences in heart rate and body temperature may have been determined by different levels of somatomotor activity, which indeed resulted significantly higher in knockout mice during the active phase of the daily cycle. However, given that heart rate was consistently higher in FTO deficient mice even when somatomotor activity levels were not greater, we believe that autonomic mechanisms concurred to determine higher heart rate in these animals. Supporting this view, HRV analysis revealed that knockout mice were characterized by a lower vagal modulation of heart rate (RMSSD and HF indexes) than wild-type counterparts. In addition, the fact that FTO deficient mice showed higher LF to HF ratio (index of sympatho-vagal balance) is suggestive of a larger contribution of the sympathetic modulation of heart rate in mice lacking the FTO gene. Signs that link FTO deficiency to increased cardiac sympathetic drive were evident during stress conditions. Following the injection of saline and during the restraint test, stress-induced tachycardia was greater in knockout mice, despite similar low levels of vagal modulation (RMSSD and HF indexes) between the two groups. This is a clear indication of a larger sympathetic modulation of heart rate in FTO deficient mice, which consequently resulted in a shift of the sympatho-vagal balance towards an exaggerated sympathetic prevalence (i.e., increased LF to HF ratio). Given that high expression of FTO is seen in the paraventricular and dorsomedial nuclei of the hypothalamus [6], [7], [10], which represent important brain centers for the regulation of autonomic function, especially during stress response [11], [12], we hypothesize a role of FTO in these brain areas in modulating sympathetic outflow to the heart.

Previous studies have demonstrated that β-adrenergic agonists increase the inward sodium current in cardiomyocytes [27]–[29]. Because the sodium current is a major determinant of conduction, it is thus reasonable to speculate that enhanced cardiac sympathetic tone is responsible for the reduction in the duration of P wave (index of atrial activation interval), PQ segment (index of atrio-ventricular conduction) and QRS complex (index of ventricular activation) within the heart of FTO deficient mice.

The sympathetic nervous system is known to play an important role in arrhythmogenesis [30]. Catecholamines can increase automaticity [31] and induce triggered activity [32], [33], thereby increasing arrhythmic risk. Importantly, in this study we provide evidence of increased vulnerability to stress-induced tachyarrhythmias in mice lacking the FTO gene. Of note, arrhythmogenesis was almost completely absent in wild-type mice. It is interesting that arrhythmia vulnerability in FTO deficient mice was (i) induced by stress exposure, as arrhythmic events were only sporadically noted during baseline recordings, and (ii) clearly more pronounced in response to the restraint than the injection stress. Taken together these findings point to a close link between FTO deficiency and arrhythmia vulnerability, particularly in conditions of sustained stress exposure (restraint stress) [34].

Investigation of potential electrophysiological changes relevant to arrhythmogenesis in the heart of FTO deficient mice revealed that no changes occurred in ventricular excitability and refractoriness, suggesting that arrhythmia vulnerability may not be linked to cellular electrophysiological abnormalities. Noteworthy, these measures were obtained in anesthetized mice (i.e., under this condition sympathetic tone is greatly suppressed [35]), and therefore we cannot exclude that sympathetic hyperactivity in FTO deficient mice may have affected cardiac excitability and/or refractoriness in the awake state. In addition, our data indicate that arrhythmogenesis was not correlated to accumulation of fibrotic tissue in the left ventricular myocardium. Our hypothesis is that exaggerated sympathetic stress response triggered abnormal automaticity in non-pacemaker tissue. We found in FTO deficient mice signs of cardiac hypertrophy, affecting both the right and the left ventricles. The specific morphological changes were not investigated here, but might reflect structural changes in the hypertrophied myocardium altering the ion channels operating during the early repolarization phase. This hypothesis was based on the observation that the duration of QTc interval (marker of ventricular repolarization) was longer in FTO deficient mice. Therefore, we hypothesize a role of ventricular hypertrophy in altering ventricular repolarization to explain QTc lengthening in these mice [36]. The QTc interval is also influenced by the autonomic nervous system: abnormal sympathetic modulation [37] or vagal withdrawal [38] directly induce altered ventricular repolarization, thus leading to prolongation of the QTc interval. Therefore, exaggerated cardiac sympathetic predominance in mice lacking the FTO gene could contribute directly to both ventricular hypertrophy and abnormal ventricular repolarization independent from blood pressure [39], conditions that might serve as a substrate for arrhythmias [40], [41]. Further studies are needed in order to elucidate the biophysical mechanisms and the cellular and subcellular bases of the reported arrhythmogenesis.

### Conclusion and Perspective

Previous studies have demonstrated that FTO deficiency in mice results in a lean phenotype [1], [13], [42]. This observation has prompted researchers to hypothesize that inhibition of FTO might be of therapeutic interest in relation to morbid obesity. Putative mechanisms underlying the lean phenotype of FTO deficient mice may include an increase in sympathetic nervous system activity, thereby promoting lipolysis and thermogenesis in adipose tissue and muscle [1], [13].

In our mouse model, FTO deficiency led to an exaggerated sympathetic contribution of the autonomic neural modulation of cardiac function and to a potentially proarrhythmic remodeling of the myocardium. We did not determine whether such autonomic imbalance in the sympathetic direction was mediated directly by hypothalamic mechanisms or indirectly by alternative mechanisms that may have occured in FTO deficient mice during development. This represents the major limitation of this study. Further investigations using brain specific and inducible FTO deficiency or FTO deficiency tied for example to certain hypothalamic (e.g. CRH) neurons may be useful for (i) revealing the precise neurobiological pathways underlying the autonomic phenotype of FTO deficient mice and (ii) determining whether reducing the expression or inactivating catalytic activity of FTO might represent a promising strategy to purse in order to alleviate obesity.
